# Simultaneous Localization and Mapping Methods for Snake-like Robots Based on Gait Adjustment

**DOI:** 10.3390/biomimetics9110710

**Published:** 2024-11-19

**Authors:** Chaoquan Tang, Zhipeng Zhang, Meng Sun, Menggang Li, Hongwei Tang, Deen Bai

**Affiliations:** Jiangsu Key Laboratory of Mine Mechanical and Electrical Equipment, School of Mechatronic Engineering, China University of Mining and Technology, Xuzhou 221116, China; tangchaoquan@cumt.edu.cn (C.T.); ts24050084a31@cumt.edu.cn (Z.Z.); dundsm@163.com (M.S.); sallylmg@cumt.edu.cn (M.L.); thw@cumt.edu.cn (H.T.)

**Keywords:** snake robot, SLAM, typical gait, stability control, IMU

## Abstract

Snake robots require autonomous localization and mapping capabilities for field applications. However, the characteristics of their motion, such as large turning angles and fast rotation speeds, can lead to issues like drift or even failure in positioning and map building. In response to this situation, this paper starts from the gait motion characteristics of the snake robot itself, proposing an improved gait motion method and a tightly coupled method based on IMU and visual information to solve the problem of poor algorithm convergence caused by head-shaking in snake robot SLAM. Firstly, the adaptability of several typical gaits of the snake robot to SLAM methods was evaluated. Secondly, the serpentine gait was selected as the object of gait improvement, and a head stability control method for the snake robot was proposed, thereby reducing the interference of the snake robot’s motion on the sensors. Thirdly, a visual–inertial tightly coupled SLAM method for the snake robot’s serpentine gait and Arc-Rolling gait was proposed, and the method was verified to enhance the robustness of the visual SLAM algorithm and improve the positioning and mapping accuracy of the snake robot. Finally, experiments proved that the methods proposed in this paper can effectively improve the accuracy of positioning and map building for snake robots.

## 1. Introduction

Currently, robots have a wide range of applications in many fields, such as security, search and rescue, and transportation. Operating in narrow and confined spaces is one of the practical requirements for robots in these fields, requiring specialized capabilities such as compact size and strong environmental adaptability [1]. Among all types of robots, serpentine robots have a thin cross-section and an elongated flexible body, which helps the body to move forward in very narrow spaces with minimal interference to the surrounding environment, making it highly advantageous in the detection of complex and narrow environments, such as post-disaster rescue, underground exploration, and so on [2]. However, in GPS denial environments, snake robots are unable to obtain their position and environmental information, making it difficult for them to realize autonomous navigation and thus difficult to discover the purpose of performing tasks autonomously [3]. SLAM technology addresses the problem of recovering the relative spatial relationship between the observer itself and its surroundings, giving a description of the surrounding environment. With this foundation, the snake robots can carry out path planning, detect obstacles encountered promptly, and avoid obstacles encountered in time [4,5].

SLAM is an important technique to estimate the state of the target and its surroundings [6,7,8]. Currently, snake robots face many challenges in SLAM applications, such as large hardware size, high energy consumption, algorithmic complexity, and high computational effort [9]. Although LiDAR and ultrasonic sensors can provide environmental depth information to help navigation and localization, they usually have insufficient depth resolution and lack color information, which limits their application in target detection and recognition [10]. In contrast, vision cameras can capture rich scene details and have the advantages of small size, low energy consumption, and cost-effectiveness, making them ideal for SLAM solutions for snake robots [11].

However, the snake robot itself has a variety of motion forms, such as serpentine, rolling, side-shifting, side-rolling, lifting up, overcoming obstacles, etc. During the rapid movement and rotation of the snake robot, there are motion characteristics such as violent shaking, large corners, and fast rotation speed that occur as a result of feature-point tracking failure, leading to poor localization and mapping accuracy, poor robustness, and even failure [12,13]. In a previous work, Gong. C et al. [14] used a modal decomposition method to keep the head of a snake robot in the same direction during rolling gait. This method ensures that the head camera of the snake robot works properly during rolling gait. H. Yamada et al. [15] stabilized the head of the snake robot by controlling the movement of its neck. However, when the method is converted from a continuous model to a discrete model, it causes the head position to move back and forth, which affects the control of the robot. Z. Bing [16] compensated for the head offset angle of the snake robot by calculating the derivative of the snake curve concerning time. Wu et al. [17] introduced virtual joints as the desired directions and analyzed the virtual joints in the HNC (Head Navigational Control) about the first two head joint angle relationships. However, this lacks validation of the head navigation effect, and it is difficult to adjust the parameters using a cyclic inhibition model. G. Qiao et al. [18] implemented partial control by proposing to divide the snake robot into different parts such as the head, neck, and torso. The torso is used to propel the snake robot forward, and the neck and head are used to control the direction of the snake robot. However, this method requires multiple modules to be treated as necks, thus increasing the number of joints and making it difficult to apply to curvature control-based CPG models. Wu et al. [19] used virtual joints based on a CPG network to achieve head stabilization. Although this scheme can effectively ensure the head stabilization of the snake robot, it lacks the feedback of head position information and is not efficient.

The low viewing angle of snake robots makes them susceptible to obstacles, which leads to visual loss and trajectory offset. Kazunori Ohno et al. [20] implemented snake robot trajectory estimation and SLAM reconstruction using an SR-2 RGBD camera and a gyroscope, which has a large yaw angle error, large size, and high power consumption. Liu et al. [21] proposed a bi-directional long short-term memory (BiLSTM) neural network for a snake robot trajectory prediction method and visual localization system, which solves the problem of the visual tracking loss of the snake robot. Kim et al. [22] proposed an error-based adaptive robust RBF-NN-BSC for effective image data reading of the snake robot head system while the snake robot is traveling. Li et al. [23] proposed a system based on the ORB-SLAM3 for stabilized RGB-D SLAM to improve localization accuracy in dynamic environments. Wang et al. [24] develop Dym-SLAM for reconstructing 4D dynamic scenes using rigid moving objects. Despite their contributions, existing methods often face challenges related to generalization, scene awareness, or accurate segmentation.

To solve the above problems, this paper starts from the typical gait motion characteristics of a snake robot, analyzes the gait characteristics of a snake robot and the adaptability with SLAM, and based on this, puts forward a scheme to improve the visual SLAM localization accuracy of the snake robot and the effect of constructing a map: by controlling the joint angle of the motion of the snake robot to improve the form of the snake robot’s movement and enhance the stability of the snake robot in the process of movement and thus improve the SLAM effect. On the other hand, from the point of view of the accuracy and robustness of the SLAM algorithm itself, the multi-sensor fusion method is used to enhance the robustness of the visual SLAM algorithm.

The overall structure of this paper is as follows: Section 2 analyzes the adaptability of each gait of the snake robot to the SLAM method. Section 3 proposes the head stability algorithm of the snake robot to reduce the amplitude of sensor shaking based on serpentine gait. Section 4 details a vision-inertial SLAM approach for snake robot motion swaying. Finally, Section 5 presents the snake robot platform for the related experiments and validates the algorithms proposed.

## 2. Adaptability Study of SLAM for Typical Gaits of Snake-like Robots

Gait generation methods are used to determine the form of motion of the snake robot by controlling the change in joint angles, while the control function method is currently the main method for controlling the gait generation of snake robots, which is characterized by model independence and flexible adjustability. The parameterized gaits are all based on sinusoidal waves in two mutually perpendicular planes: a horizontal plane parallel to the ground and a vertical plane perpendicular to the ground and parallel to the snake’s longitudinal axis. When the wave perpendicular to the ground is sent, only the even-numbered joints are involved in the motion and the odd-numbered joints remain stationary; similarly, only the odd-numbered modules are involved in the motion of the transverse wave sent through the robot parallel to the ground [25]. Each joint angle is modeled as a sinusoidal function with a phase shift by describing the gait through joint angles. Then, the angle of the *n*th joint at the moment t can be expressed as follows:

(1) $$\alpha (n,t)=\left\{\begin{array}{l} \beta_{odd}+A_{odd}\sin(\theta_{odd}),n=odd\\ \beta_{even}+A_{even}\sin(\theta_{even}+\delta ),n=even \end{array}\right.$$

(2) $$\theta =\frac{d\theta }{dn}n+\frac{d\theta }{dt}t$$

where β is the offset, A is the amplitude, θ is the frequency, δ is the phase shift, $A_{even}$ and $A_{odd}$ is the amplitude term, dθ/dn determines the shape of the serpentine robot, and dθ/dt is the magnitude of the frequency.

Based on the above model, the motion adaptations of four typical gaits, namely, serpentine gait, rolling gait, pedal gait, and sidewinding gait, are analyzed sequentially for ORB-SLAM.

The meandering gait, as the most common two-dimensional locomotion for snake-like robots, is suitable for movement on flat surfaces, propagating body waves from the head to the tail, which is the most widespread mode of movement exhibited by biological snakes. The rolling gait is unique to snake-like robots, including two-dimensional arc rolling within a plane and three-dimensional helix rolling in space, which is not a natural movement for biological snakes. In the pedal gait, the robot makes contact with the ground at only a few points to generate thrust while lifting areas with high curvature to reduce friction. The sidewinding gait combines transverse and longitudinal waves, allowing the robot to both flip and side-shift perpendicular to the body direction.

The form of snake robot motion under serpentine gait is sinusoidal, so the size of the sinusoidal wave amplitude will be an important factor affecting the adaptability of this gait with ORB-SLAM, while the increase in speed will make the snake robot’s left and right swing frequency speed up. Therefore, the idea of studying the adaptability of testing the serpentine gait with ORB-SLAM is to change the wave amplitude of the snake robot’s motion and the speed of the snake robot’s motion under each wave amplitude. Based on the analysis of the error between the estimated value and the true value of the motion, the effect of the size of the wave amplitude and the speed of the motion on the SLAM adaptability of the snake robot is judged.

The snake robot model for the adaptive analysis in this section consists of 35 joints with a length of 89.95 mm, and the robot model’s joints are connected orthogonally to each other. Since amplitude A is related to α, the change in parameter A is replaced by changing α. When the parameters of the fitting curve are five joint lengths and α is 0.1π, the wave amplitude is small during the motion of the snake robot, and the maximum pendulum amplitude is 0.173 m (5.50%). The head stability of the snake robot in this state is better, the feature extraction is continuous, and there is no tracking loss. When α = 0.3π, the swing amplitude of the snake robot increases significantly, with a maximum swing amplitude of 0.416 m (13.2%) and the SLAM method estimates the trajectory to be cluttered during the simulation, then the SLAM method is assumed to be invalid. When α = 0.4π, 0.5π, even if the fitting curve parameter is set to a joint length, it is still impossible to build a map, and the maximum swing is 0.56 m (17.8%) when α = 0.4π, and 0.62 m (19.7%) when α = 0.5π. As the parameter increases, the waveforms of the motion become bigger and bigger, which leads to less or even no feature extraction, and the visual front-end cannot obtain data correlation through feature matching, which leads to the failure of camera position solving.

Under the arc-rolling gait, the snake robot rotates around its axis. Therefore, the idea of testing the localization effect of the snake robot under this gait is to change the size of the fitting curve parameters while keeping other parameters unchanged. Through the simulation analysis, the arc-rolling gait movement speed is lower, the image captured by the camera is clearer, at this time the feature extraction is more stable, and there is no loss of keyframes; pure rotary motion lacks translation-related data excitation, the motion matrix degrees of freedom remain unchanged, while the motion itself decreases in the degrees of freedom, which makes the extra degrees of freedom susceptible to the influence of noise; with the increase in speed, the image captured by the camera appears to be dragged, which is a good idea. With the increase in speed, the image captured by the camera suffers from the phenomenon of dragging, leading to a reduction in the number of features extracted, and the system suffers from the problem of lost trajectory tracking due to insufficient features, which in turn leads to the failure of the ORB-SLAM scheme.

Due to the characteristics of the pedal gait, the snake robot sacrifices stability characteristics while pursuing high motion efficiency. In the vision SLAM method with ORB features as the front-end, the pedal gait causes the camera attached to the head of the snake robot to shake up and down as the snake robot jerks up and down drastically during the motion process, which leads to the failure of the extraction of the ORB feature points and the matching of the failure of the feature. The ORB-SLAM scheme fails in this gait.

In comparison with the arc-rolling gait, the rotation of the sidewinding gait is a rotation around the support point with the surface at different moments rather than a rotation around its axis. The head joints of the snake robot cannot operate smoothly in this gait because the snake robot undergoes a large displacement at the same time as the rotation. The camera shoots the ground for a long time in this gait, and the ORB feature extraction fails due to the lack of texture information and the high speed. So the ORB-SLAM scheme fails in this gait.

From the above analysis, it can be concluded that the pedal gait and the sidewinding gait are poorly adapted to SLAM due to high-frequency jerks and are not suitable for performing the tasks of localization and map building. Therefore, the sidewinding gait and the arc-rolling gait are selected as SLAM research objects. To improve the SLAM adaptability of the snake robot gait, several methods are proposed to address the problems of the large swings of the head joints and the too fast pure rotational speed in the movement process.

## 3. Research on Visual SLAM Method Based on Head Stability Control of Snake-like Robots

For the snake robot’s motion gait on the horizontal plane, which refers to the robot’s movement within the two-dimensional space defined by the ground surface, the serpentine gait makes head stability control a possibility because of the smooth gait motion characteristics. Therefore, the serpentine gait is selected as the research object to improve head stability during its motion, which in turn improves the accuracy and stability of the visual SLAM during the serpentine gait.

### 3.1. Modeling of Head Stability Control Based on Link Angle Correction

**Figure 1:**
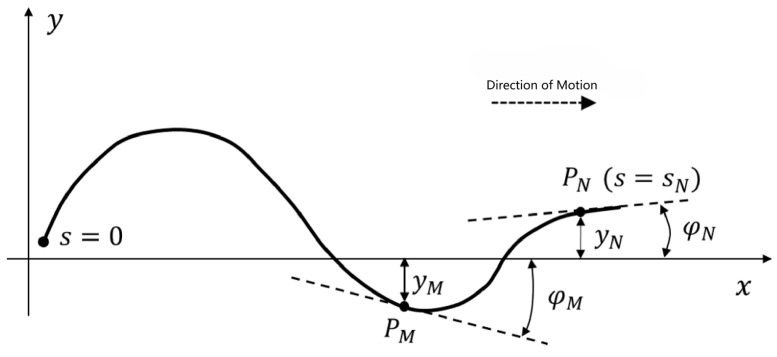
Principle of head stability control.

The snake robot consists of three parts: the “body”, “neck”, and “head”. It is assumed that when the snake robot carries out the serpentine movement, the “body” carries out the undulating movement. During the process, the “head” and “neck” parts are influenced by the fluctuation and thus perform the same undulating movement as the “body”. Therefore, the key to the control principle is to find a way to control the “neck” to eliminate the effect of the “body” fluctuation and stabilize the “head”. Figure 1 shows the model of the snake robot, where the X-axis represents its direction of movement and the Y-axis represents the left side of the movement.

Where $P_{M}$ is the point of demarcation between the “cervical region” and the “neck”, and $P_{N}$ is the boundary point between the “head” and the “neck”.

To realize the stability control of the motion direction and position of the snake robot that is equivalent to satisfy $\overset{•}{\varphi_{N}}\left(t\right)=0$ and $\overset{•}{y_{N}}\left(t\right)=0$. At this moment, the head of the snake robot is kept in a neutral position, located on the x-axis, namely, the forward direction of the snake robot.

### 3.2. Head Direction Stability Control

**Figure 2:**
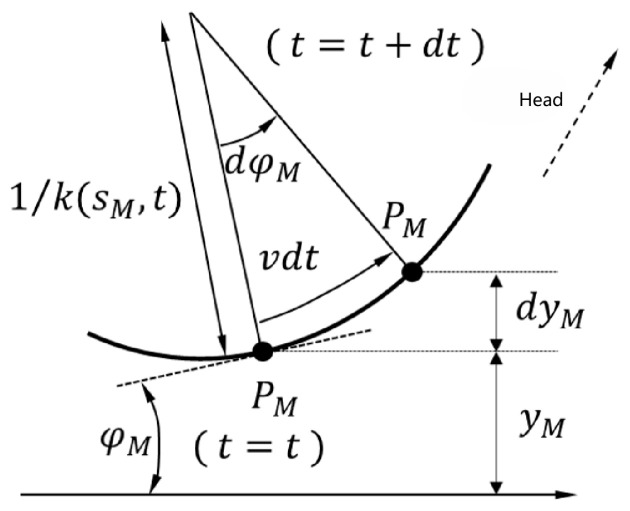
Angular differential variation.

The head direction stabilization control method ensures that the head of the snake robot points in one direction as much as possible, rather than constantly and drastically changing direction as the snake swings. Assuming that the motion of point $P_{M}$ of the serpentine robot is as shown in Figure 2. $P_{M}$ is moving around a curve of radius of 1/k(s,t), and we can obtain the following:

(3) $$\overset{•}{\varphi_{M}}(t)=k(s_{M},t)\cdot v$$

where $\overset{•}{\varphi_{M}}(t)$ is the angle between the X-axis and the tangent line at $P_{M}$, $s_{M}$ is the arc length at $P_{M}$, k(s,t) is the curvature at s=s,t=t, and v is the velocity at moment t.

It is assumed during the motion of the snake robot that the snake robot will not slide on the y-axis during its motion, which means that there will be no rotational motion due to sliding. Since the parameters on the right side of the equation are controlled by the motion of the snake robot, we obtain the following:

(4) $$\varphi_{N}(t)-\varphi_{M}(t)=\varphi (s_{N},t)-\varphi_{M}(s_{M},t)=\int_{s_{M}}^{s_{N}}k(s,t)ds$$

Differentiating Equation (4) and shifting the terms yields Equation (5).

(5) $$\overset{•}{\varphi_{N}}(t)=\overset{•}{\varphi_{M}}(t)+\int_{s_{M}}^{s_{N}}k(s,t)ds$$

To satisfy head direction stability, namely, make $\overset{•}{\varphi_{N}}\left(t\right)=0$, $\overset{•}{\varphi_{M}}(t)$ can be obtained as follows:

(6) $$\overset{•}{\varphi_{M}}(t)=-\int_{s_{M}}^{s_{N}}k(s,t)ds$$

Using Equations (3) and (6), then Equation (7) can be expressed as follows:

(7) $$\int_{s_{M}}^{s_{N}}k(s,t)ds=-k(s_{M},t)\cdot v$$

Equation (7) is the control theory for the head stability of the snake robot in the serpentine gait described in this chapter. From Equation (4), when fitting the head direction of the snake robot to the motion direction at the beginning of the motion, k(s,t) should also satisfy the equation as follows:

(8) $$\int_{s_{M}}^{s_{N}}k(s,t)ds=-\varphi_{M}(t)$$

Therefore, for simplicity of calculation, it is assumed that when $s_{M}<s<s_{N}$, the curvature is a constant, namely $k(s,t)=c_{M}(t)$. The “neck” is a circular arc in this case. At any moment, the arc of the circle and the body part of the sine wave articulation can point the direction of the head of the snake robot to the direction of its movement. According to the curvature of the arc, to solve the formula, the ratio of the angle of the center of the circle to the length of the arc and combined with Equation (7) can be seen when Equation (9) is valid and is then a constant. The $\overset{•}{c_{M}}(t)$ is expressed as follows:

(9) $$\overset{•}{c_{M}}(t)=-\frac{k(s_{M},t)}{l_{M}}t$$

Thus, the curvature of the snake curve in serpentine gait is obtained as a segmented function:

(10) $$k(s,t)=\left\{\begin{array}{l} -\alpha b\sin(bs+\sigma (t)),s<s_{M}\\ -\frac{\varphi_{M}(t)}{l_{N}},s_{M}<s<s_{N} \end{array}\right.$$

where b=2π/L. Finally, the mathematical formulation for controlling the head stability of the snake robot in the serpentine gait is obtained by integration as follows:

(11) $$\varphi (s,t)=\left\{\begin{array}{l} \alpha \cos(bs+\sigma (t)),s<s_{M}\\ (1-\frac{s-s_{M}}{l_{M}})\alpha \cos(bs+\sigma (t)),s_{M}<s<s_{N} \end{array}\right.$$

where σ(t) is the function of time.

From Equation (9), the joint position of the *i*th joint can be expressed as follows:

(12) $$Angle=\varphi ((2i)\cdot l_{0})-\varphi ((2i-2)\cdot L_{0})$$

### 3.3. Head Position Stability Control

The head position stabilization control method can build on the algorithm in the previous section to minimize the sensor wobble caused by the snake robot’s head swinging from side to side during its serpentine motion. As shown in Figure 2, point $P_{M}$ of the “neck” moves with speed v in the direction of the tangent point M. The case of satisfying the assumptions made in the previous section, $\overset{•}{y_{M}}(t)$ can be expressed as follows:

(13) $$\overset{•}{y_{M}}(t)=-\sin\varphi_{M}(t)\cdot v$$

Substitute Equation (6) into Equation (13).

(14) $$y_{N}(t)=y_{M}(t)+\int_{s_{M}}^{s_{N}}\sin(k(s,t)ds+\varphi_{M}(t))ds$$

Derivate Equation (14) and substitute Equation (6) into Equation (13).

(15) $$\int_{s_{M}}^{s_{N}}\sin(\int_{s}^{s_{H}}k(s,t)ds)ds=-\sin(\int_{s_{M}}^{s_{N}}k(s,t)ds)\cdot v$$

From the above derivation, it can be seen that any curvature satisfying Equation (15) can stabilize the position and direction of the head of the snake robot, but the complexity of Equation (15) leads to the problem of complicated and difficult curvature calculation. In the previous subsection, to simplify the calculation, it is assumed that the “neck” of the snake robot is a circular arc. So it is assumed that the snake robot consists of two circular arcs with openings at both ends in the opposite direction, and Equation (15) is linearized. Thus, when $s>s_{M}$, the curvature expression is divided into two parts:

(16) $$k(s,t)=\left\{\begin{array}{l} c_{M}(t)+f_{M}(t),s_{M}<s<s_{M}+l_{N}/2\\ c_{M}(t)-f_{M}(t),s>s_{M}+l_{N}/2 \end{array}\right.$$

Substitute Equation (15) into Equation (16). The differential expression for f(t) can be expressed as follows:

(17) $$\overset{•}{f_{M}}(t)=\frac{4c_{M}(t)}{l_{N}}-2\frac{k(s_{M},t)}{l_{N}}$$

Integrating Equation (17) yields an expression for the curvature. To facilitate the computation of the curvature, in this paper we use the discretization method to solve f(t).

(18) $$f_{M}(t_{k+1})=f_{M}(t_{k})+\overset{•}{f_{M}}(t_{k})\cdot \delta t$$

Finally, the curvature of the head direction and position stability of the snake robot in the serpentine gait is obtained into three segments as in Equation (19).

(19) $$k(s,t)=\left\{\begin{array}{l} -\alpha b\sin(bs+\sigma (t)),s<s_{M}\\ c_{M}(t)+f_{M}(t),s_{M}<s<s_{M}+l_{N}/2\\ c_{M}(t)-f_{M}(t),s>s_{M}+l_{N}/2 \end{array}\right.$$

Integrate over the curvature. φ(s,t) can be expressed as follows:

(20) $$\varphi (s,t)=\left\{\begin{array}{l} \alpha \cos(bs+\sigma (t)),s<s_{M}\\ (1-\frac{s-s_{M}}{l_{N}})\alpha \cos(bs+\sigma (t))+f_{M}(t)(s-s_{M}),s_{M}<s<s_{M}+l_{N}/2\\ (1-\frac{s-s_{M}}{l_{N}})\alpha \cos(bs+\sigma (t))+f_{M}(t)(s-s_{M}-l_{N}/2),s>s_{M}+l_{N}/2 \end{array}\right.$$

Similarly, the joint position of the *i*th joint can be obtained as follows:

(21) $$Angle=\varphi ((2i)\cdot l_{0})-\varphi ((2i-2)\cdot L_{0})$$

## 4. Research on Visual–Inertial Tightly Coupled SLAM Method for Snake-like Robots

From the analysis in Section 2, it can be seen that the snake robot’s serpentine gait and arc-rolling gait suffer from poor head stability due to large head joint swing and large SLAM drift due to too fast rotation speed, respectively. In this chapter, we will improve the adaptability of the snake robot’s typical gait with SLAM from the perspective of improving the robustness of the algorithm itself. Inertial Measurement Units (IMU) complement the vision sensors well, so this chapter will incorporate new sensor-IMUs within the vision SLAM framework to act as a constraint on the vision estimation [26,27]. Firstly, the vision-inertial SLAM is used as an observation; then, the kinematic models of the snake robot’s serpentine gait and arc-rolling gait are utilized as the motion models, and finally, the IESKF algorithm is utilized for estimating the final results, thus improving the robustness of the algorithm.

### 4.1. Visual–Inertial-Based Observation Models

When the camera is only the sensor of the SLAM system, the surrounding waypoints will only constrain the camera, and at this time, it is only necessary to construct the reprojection error as a least-squares problem to find the optimal solution. However, a trajectory will also be generated based on the IMU data as well as the equations of motion as the carrier is in motion when an IMU is added. Since both the camera and the IMU are solidly attached to a carrier, there is a connection between the trajectory information estimated by each of the two sensors, and this connection can be determined by the external reference between the camera and the IMU.

The sampling frequency of the IMU is higher than that of the camera, and the bit position estimated by the camera is usually the bit position between the keyframes, so the IMU observations between two frames need to be accumulated. When constructing the optimization problem of the vision-inertial system, the visual information, along with the IMU zero-bias, velocity, and gravity direction, need to be used as the optimization vectors, and while iteratively updating these vectors, the information of the key frames’ position, velocity, and so on, will continuously be performed as integration operations, which wastes the computational resources, and therefore, the pre-integration of the IMU is needed, i.e., the terms that are invariant at each iteration of the optimization are extracted, and the PVQ is obtained for each cycle. Position, velocity, and attitude increments are measured against other estimates of PVQ increments obtained by non-IMU means, and then the residuals of the PVQ increments are obtained. This residual is then used to construct a cost function to iteratively update and optimize the PVQ for each node.

The measured values of the gyroscope and accelerometer are first constructed as follows:

(22) $$\begin{array}{l} \omega_{M}(t)=\omega_{b}(t)+b_{g}(t)+n_{g}\\ a_{M}(t)=a_{b}(t)+b_{a}(t)+R_{w}^{b_{t}}g^{w}+n_{a} \end{array}$$

where $\omega_{m}\left(t\right)$ is the angular velocity measurement of the gyroscope at moment t, $\omega_{b}\left(t\right)$ is the true value of the gyroscope angular velocity at moment t, $b_{g}(t)$ is the random zero bias of the gyroscope at moment t, $n_{g}$ is the gyroscopic white noise, $a_{m}\left(t\right)$ is the measured acceleration of the gyroscope at moment t, $a_{b}(t)$ is the true value of the acceleration of the gyroscope at moment t, $b_{a}\left(t\right)$ is the random zero bias of the accelerometer at moment t, $R_{w}^{b_{t}}$ is the rotation matrix for conversion from the world coordinate system to the IMU coordinate system, $g^{w}$ is the acceleration in the world coordinate system, and $n_{a}$ is the accelerometer white noise.

Integrate the PVQ of frame k, and the PVQ of frame (k + 1) can be expressed as follows:

(23) $$\begin{array}{l} p_{b_{k+1}}^{w}=p_{b_{k}}^{w}+v_{b_{k}}^{w}\Delta t_{k}+\iint_{t\in [t_{k},t_{k+1}]}\{R_{b_{t}}^{w}[a_{m}(t)-b_{a}(t)-n_{a}]-g^{w}\}dt^{2}\\ v_{b_{k+1}}^{w}=v_{b_{k}}^{w}+\int_{t_{k}}^{t_{k+1}}\{R_{b_{t}}^{w}[a_{m}(t)-b_{a}(t)-n_{a}]-g^{w}\}dt\\ q_{b_{k+1}}^{w}=q_{b_{k}}^{w}⊗\int_{t_{k}}^{t_{k+1}}\frac{1}{2}\Omega (\omega_{m}(t)-b_{g}(t)-n_{g})q_{t}^{b_{k}}dt \end{array}$$

where w is the world coordinate system, $b_{k}$ and $b_{k+1}$ are the different image sampling moments, ⊗ denotes the operations between rotating quaternions, and $q_{t}^{b_{k}}$ denotes the transformation matrix of the position from moment t to moment $b_{k}$.

(24) $$\Omega (\omega )=\left[\begin{array}{cc} -{\left[\omega \right]}_{\times } & \omega \\ -\omega^{T} & 0 \end{array}\right],{\left[\omega \right]}_{\times }=\left[\begin{array}{ccc} 0 & -\omega_{z} & \omega_{y}\\ \omega_{z} & 0 & -\omega_{x}\\ -\omega_{y} & \omega_{x} & 0 \end{array}\right]$$

Perform the left-multiplication of Equation (24).

(25) $$\begin{array}{l} R_{w}^{b_{k}}p_{b_{k+1}}^{w}=R_{w}^{b_{k}}(p_{b_{k}}^{w}+v_{b_{k}}^{w}\Delta t_{k}-\frac{1}{2}g^{w}\Delta t_{t_{k}}^{2})+\alpha_{b_{k+1}}^{b_{t}}\\ R_{w}^{b_{k}}v_{b_{k+1}}^{w}=R_{w}^{b_{k}}(v_{b_{k}}^{w}-g^{w}\Delta t_{k})+\beta_{b_{k+1}}^{b_{k}}\\ q_{w}^{b_{k}}⊗q_{b_{k+1}}^{w}=\gamma_{b_{k+1}}^{b_{k}} \end{array}$$

where $\alpha_{b_{k+1}}^{b_{k}}$, $\beta_{b_{k+1}}^{b_{k}}$, and $\gamma_{b_{k+1}}^{b_{k}}$ are pre-integral, and the expression can be expressed as follows:

(26) $$\begin{array}{l} \alpha_{b_{k+1}}^{b_{k}}=\iint_{t\in [t_{k},t_{k+1}]}\{R_{b_{l}}^{b_{k}}[a_{m}(t)-b_{a}\left(t\right)-n_{a}]\}dt^{2}\\ \beta_{b_{k+1}}^{b_{k}}=\int_{t_{k}}^{t_{k+1}}\left\{R_{b_{l}}^{b_{k}}[a_{m}(t)-b_{a}(t)-n_{a}]\right\}dt\\ \gamma_{b_{k+1}}^{b_{k}}=\int_{t_{k}}^{t_{k+1}}\frac{1}{2}\Omega (\omega (t)-b_{g}(t)-n_{g})\gamma_{t}^{b_{k}}dt \end{array}$$

The IMU pre-integration error term between image sampling from moment $b_{k}$ to moment $b_{k+1}$ is $r_{B}({\hat{z}}_{b_{k+1}}^{b_{k}},\chi )$

(27) $$r_{B}(\overset{\wedge }{z_{b_{k+1}}^{b_{k}}},\chi )=\left[\begin{array}{l} \delta \alpha_{b_{k+1}}^{b_{k}}\\ \delta \beta_{b_{k+1}}^{b_{k}}\\ \delta \gamma_{b_{k+1}}^{b_{k}}\\ \delta b_{a}\\ \delta b_{w} \end{array}\right]=\left[\begin{array}{c} R_{w}^{b_{k}}(p_{\delta_{k}+1}^{w}-p_{\delta_{k}}^{w}-v_{\delta_{k}}^{w}\Delta t_{k}+\frac{1}{2}g^{w}\Delta t_{k}^{2})-\alpha_{b_{k+1}}^{b_{k}}\\ R_{w}^{b_{k}}(v_{b_{k+1}}^{w}-v_{b_{k}}^{w}-g^{w}\Delta t_{k})-\beta_{b_{k+1}}^{b_{k}}\\ 2[q_{w}^{b_{k}}⊗q_{b_{k+1}}^{w}{\left(\gamma_{b_{k+1}}^{b_{k}}\right)}^{-1}]xyz\\ b_{a_{k+1}}-b_{a_{k}}\\ b_{g_{k+1}}-b_{g_{k}} \end{array}\right]$$

where $\overset{\wedge }{z_{b_{k+1}}^{b_{k}}}=\left(\alpha_{b_{k+1}}^{b_{k}},\beta_{b_{k+1}}^{b_{k}},\gamma_{b_{k+1}}^{b_{k}}\right)$ is the IMU integral term, and χ is the state variables to be optimized.

### 4.2. Snake Robot Motion Estimation

Suppose for the serpentine gait, the snake robot is equivalently composed of *N* connecting rods with length of 2l and uniform mass distribution m and connected at the head and tail. The center of mass inertia coordinates of the whole snake robot are expressed as follows:

(28) $$P=\left[\begin{array}{l} p_{x}\\ p_{y} \end{array}\right]=\left[\begin{array}{l} \frac{1}{Nm}\sum_{i=1}^{n}mx_{i}\\ \frac{1}{Nm}\sum_{i=1}^{n}my_{i} \end{array}\right]=\frac{1}{N}\left[\begin{array}{l} e^{T}X\\ e^{T}Y \end{array}\right]$$

The serpentine robot linkage joint constraints in matrix form can be expressed as follows:

(29) $$\begin{array}{l} DX+lA\cos\theta =0\\ DY+lA\sin\theta =0 \end{array}$$

where D is subtraction of matrices, $X={\left[x_{1},\ldots ,x_{n}\right]}^{T}$, $Y={\left[y_{1},\ldots ,y_{n}\right]}^{T}$, and $\theta ={\left[\theta_{1},\ldots ,\theta_{n}\right]}^{T}$. Combining Equations (28) and (29) yields Equation (30)

(30) $$\begin{array}{c} TX=\left[\begin{array}{c} -lA\cos\theta \\ p_{x} \end{array}\right]\\ TY=\left[\begin{array}{c} -lA\sin\theta \\ p_{y} \end{array}\right] \end{array}$$

where $T=\left[\begin{array}{l} D\\ \frac{1}{N}e^{T} \end{array}\right]\in R^{N*N}$, then $T^{-1}={\left[\begin{array}{c} D^{T}{\left(DD^{T}\right)}^{-1}\\ e \end{array}\right]}^{T}$.

The linear velocity of the connecting rod can be expressed as follows:

(31) $$\begin{array}{l} \overset{•}{X}=lK^{T}S_{\theta }\overset{•}{\theta }+e\overset{•}{p_{x}}\\ \overset{•}{Y}=-lK^{T}C_{\theta }\overset{•}{\theta }+e\overset{•}{p_{y}} \end{array}$$

where $S_{\theta }=diag(\sin\theta )\in R^{N*N}$, $C_{\theta }=diag(\cos\theta )\in R^{N*N}$.

According to the motion characteristics of the arc-rolling gait, the motion of the snake robot along the forward direction is divided into two parts; one is the rolling of the joints in contact with the ground, and its velocity can be expressed as follows:

(32) $$v_{wheel}=\omega \cdot r$$

where r is the radius of the robot module. The other is the forward swing of the elevated joint, the motion of which can be expressed in polar coordinates with the value of:

(33) $$\left\{\begin{array}{l} r_{l}(t)=l\cdot \sin(\frac{1}{2}\alpha (t))=l\cdot \sin(\frac{A}{2}\cos(\omega t))\\ \theta (t)=\omega t \end{array}\right.$$

where l is the length of the robot joint. Then, the oscillating velocity of the joint in the x-axis and z-axis can be expressed as follows:

(34) $$\left\{\begin{array}{l} v_{swing}^{x}=\frac{dx}{dt}=\frac{d}{dt}(r_{l}(t)\cdot \cos\theta (t))\\ v_{swing}^{z}=\frac{dz}{dt}=\frac{d}{dt}(r_{l}(t)\cdot \sin\theta (t)) \end{array}\right.$$

The velocity of motion of the joint along the target direction is summarized as follows:

(35) $$v(t)=\left\{\begin{array}{l} v_{wheel}+v_{swing}^{x},t\in (0,T/4]\\ v_{wheel},t\in (T/4,T/2] \end{array}\right.$$

Then, the explicit expression for the motion velocity of the robot’s joint points can be obtained as follows:

(36) $$\begin{array}{c} v(t)=\omega \cdot r+\omega \cdot l\cdot [\frac{A}{2}\cdot \cos(\frac{A}{2}\cos(\omega t))\cdot (-\sin(\omega t))\cdot \cos(\omega t)\\ +\sin(\frac{A}{2}\cos(\omega t))\cdot (-\sin(\omega t))] \end{array}$$

### 4.3. State Fusion Estimation for Snake Robots Based on IESKF

First, we define the state vector of the snake robot, which can be expressed as follows:

(37) $$x={\left[p_{x},p_{y},\theta ,v,\omega \right]}^{T}$$

where $p_{x}$ and $p_{y}$ is the coordinates of the robot’s position in a two-dimensional plane, θ is the angle of the robot’s forward direction for the global coordinate system, v is the linear velocity, and ω is the angular velocity.

Considering the motion characteristics of the snake robot, the state transfer model can be expressed as:

(38) $$x_{k+1}=f(x_{k},u_{k},\Delta t)+w_{k}$$

where f is the state transition function, $u_{k}$ is the control input, Δt is the time interval, and $w_{k}$ is the process noise.

The observation model combines data from vision and IMU and can be expressed as follows:

(39) $$z_{k}=h(x_{k})+v_{k}$$

where $z_{k}$ is the observation vector, h is the observation function, and $v_{k}$ is the observational noise.

The IESKF algorithm consists of two main steps: prediction and calibration. In the prediction step, the state at the next moment is predicted based on the current state estimation and control inputs:

(40) $$\begin{array}{l} {\overset{\wedge }{x}}_{k\left|k-1\right.}=f({\overset{\wedge }{x}}_{k-1\left|k-1\right.},u_{k-1},\Delta t)\\ P_{k\left|k-1\right.}=FP_{k-1\left|k-1\right.}F^{T}+Q \end{array}$$

In the calibration step, the state estimates are updated using the observed data:

(41) $$\begin{array}{l} y_{k}=z_{k}-h({\overset{\wedge }{x}}_{k\left|k-1\right.})\\ S_{k}=H_{k}P_{k\left|k-1\right.}H_{k}^{T}+R\\ K_{k}=P_{k\left|k-1\right.}H_{k}^{T}S_{k}^{-1}\\ {\overset{\wedge }{x}}_{k\left|k\right.}={\overset{\wedge }{x}}_{k\left|k-1\right.}+K_{k}y_{k}\\ P_{k\left|k\right.}=(I-K_{k}H_{k})P_{k\left|k-1\right.} \end{array}$$

where F is the state transfer Jacobi matrix, $H_{k}$ is the observation of the Jacobi matrix, Q is the process noise covariance matrix, R is the observation noise covariance matrix, $K_{k}$ is the Kalman gain, $y_{k}$ is the residual vector, and I is the identity matrix.

## 5. Simultaneous and Experimental Research on SLAM for Snake-like Robots

### 5.1. Simulation Verification

In this paper, we use CoppeliaSim software (V4.0.0) as the robot simulation platform and directly use CoppeliaSim to build the snake robot model. The snake robot model built in this paper consists of 35 joints with a length of 89.95 mm, and the robot model’s joints are connected orthogonally to each other.

**Figure 3:**
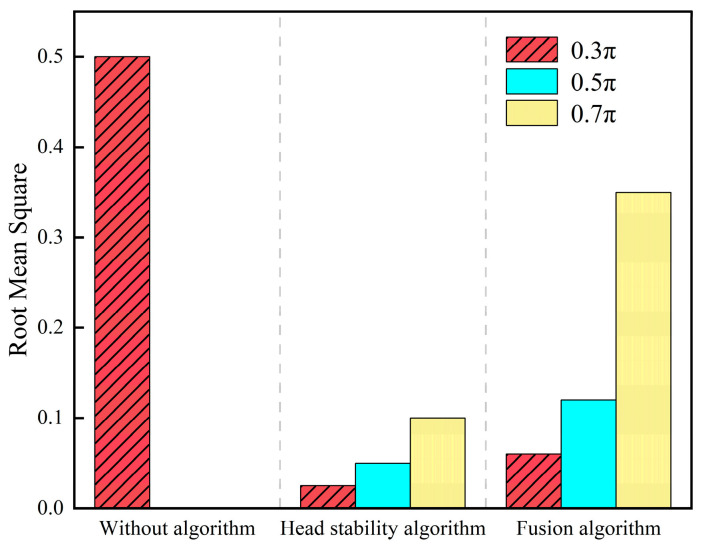
Comparison of SLAM simulation under the serpentine gait.

**Figure 4:**
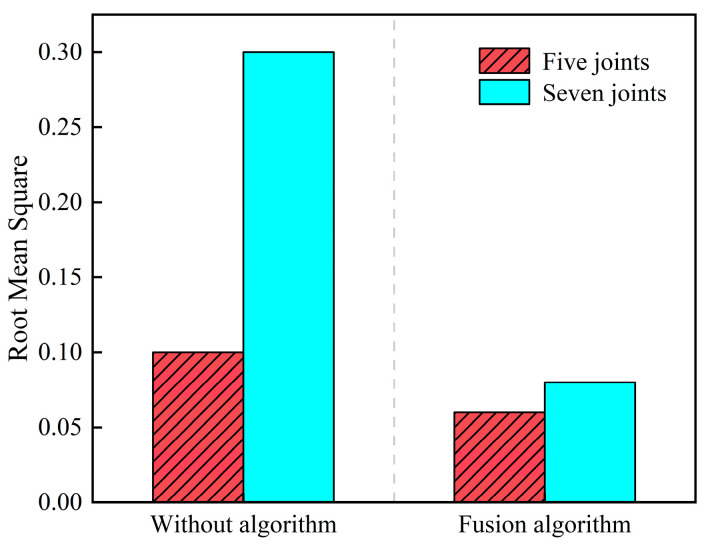
Comparison of SLAM simulation under the arc-rolling gait.

The serpentine motion simulation experiments were conducted by taking α as 0.3π, 0.5π, and 0.7π, respectively, and the comparison results of the no algorithm, stability algorithm, and fusion algorithm serpentine motion SLAM simulations are shown in Figure 3:

The arc-rolling motion simulation experiment is divided into two groups; in the case of keeping other parameters unchanged, the first group is set to five snake robot joint lengths, and the second group is set to seven joint lengths. The simulation comparison results are shown in Figure 4.

### 5.2. Experiment Verification

#### 5.2.1. Experimental Platform Introduction

**Figure 5:**
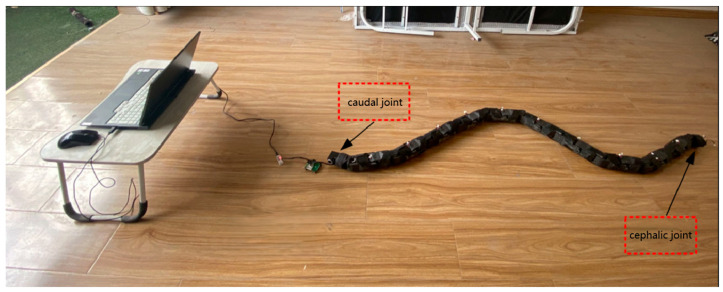
The experimental system of the snake robot.

The snake robot control system is co-built with an orthogonal snake robot as the ontological structure, an ROS open source robot system as the overall architecture, and two-level distribution of the upper and lower computers. The whole system consists of an orthogonal snake robot body, a perception module, and other hardware components. The orthogonal snake robot is alternately composed of orthogonal joints and pitch joints, with a total of 24 joints, a joint length of 96 mm, and a diameter of 64 mm. Each joint is covered with rubber to increase friction, and the prototype of the snake robot is shown in Figure 5. The sensing module mainly integrates the depth camera, core module, and battery pack. The high-resolution depth camera is used to collect image information and provide raw data for SLAM; the core module serves as the core of the perception module and provides arithmetic support for the realization of the SLAM scheme; the battery pack supplies power to the head module. Among them, the Intel RealSense l515 camera is selected as the vision sensor in this paper, which has a very small size and weight and can capture high-quality color images. The NVIDIA Jetson Xavier NX core board is used as the embedded computing platform, and the Y-C7 is used as the industrial-grade carrier board that is used in conjunction with the NVIDIA Jetson Xavier NX core module, which can capture high-quality color images. The Y-C7 is an industrial-grade carrier board for use with the NVIDIA Jetson Xavier NX core module, featuring low cost and small size.

#### 5.2.2. Experiments on Simultaneous Localization and Mapping for Snake-like Robots in the Serpentine Gait

**Figure 6:**
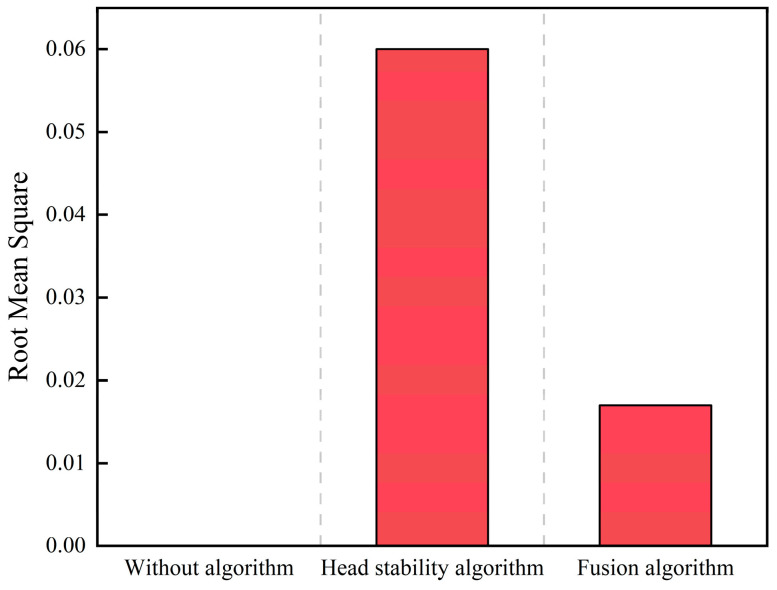
Comparison of localization and mapping under the serpentine gait.

**Figure 7:**
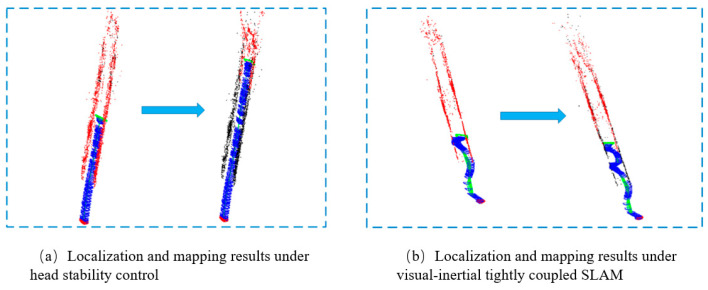
Comparison of localization and mapping under the two methods.

The experiments on simultaneous localization and mapping of the snake robot under the serpentine gait are divided into three groups of cases, namely, the condition without head stability control, the condition with head stability control added, and the vision-inertia tight coupling method. The amplitude parameter α=0.5π is set for all three groups, and the simulation analysis shows that the vision algorithm fails under this amplitude parameter. The first group of experiments can be compared with the second and third groups of experiments: the first and the second groups control the effect of the head stability control method on the improvement of the SLAM accuracy of the snake robot; the first and the third groups control the effect of the tightly coupled vision-inertia SLAM method on the improvement of the SLAM accuracy of the snake robot and the robustness of the algorithm itself. The results of the experimental comparison are shown in Figure 6 and Figure 7:

#### 5.2.3. Experiments on Simultaneous Localization and Mapping for Snake-like Robots in the Arc-Rolling Gait

**Figure 8:**
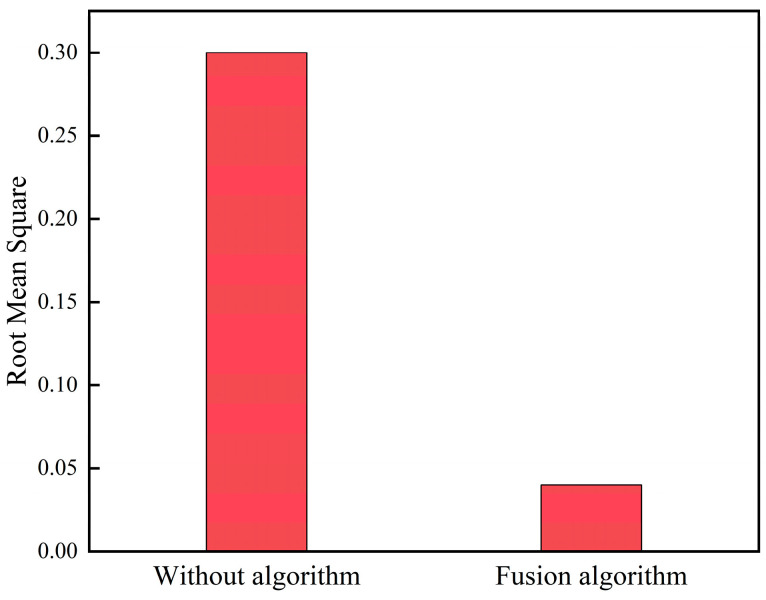
Comparison of localization and mapping under the arc-rolling gait.

**Figure 9:**
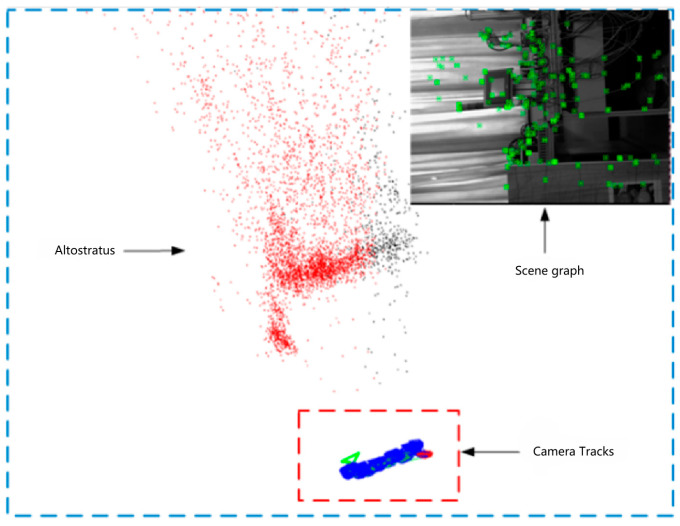
Localization and mapping results under the arc-rolling gait.

The analysis indicates that when the fitting curve parameter is 7 joint lengths, the estimated trajectory of the snake robot under the arc-rolling gait has a large drift, so this set of experiments set the fitting curve parameter of the snake robot to 7 joint lengths. The experiment is divided into two groups; in the case of keeping other parameters unchanged, the first group is set to without algorithm, and the second group is set to fusion algorithm. The experimental results are shown in Figure 8 and Figure 9:

The trajectory values show that in the arc-rolling gait, the snake robot moved 1.85 m in the x-axis direction and rotated around the axis in the y-axis direction. The mean root mean square (RMS) of the mean difference in this set of experiments is 0.04, which indicates that the snake robot has high accuracy in localization and map building under this method.

## 6. Conclusions

In this paper, we propose a SLAM method based on the head stabilization control method and the fusion of kinematic estimation of the snake robot to address the problem of the easy failure of localization and mapping caused by the large head joint angle and fast rotation speed during the motion of the snake robot, which can effectively improve the accuracy of the localization and mapping of the snake robot. Through simulation and physical experimentation, the head stability control method, under the undulating gait and with a waveform amplitude of 0.7π, achieved an average root mean square (RMS) error of 0.058 in localization and map building, significantly outperforming the SLAM scheme that fails under an amplitude of 0.3π without head stability control. The visual–inertial tightly coupled SLAM method demonstrated average root mean square (RMS) errors of 0.1 for the undulating gait and 0.08 for the arc-rolling gait, both below 0.1, indicating that this approach effectively enhances the robustness of the algorithm. The SLAM algorithm based on the head stability method can reduce the root-mean-square (RMS) of the error from 0.3 to 0.05 when the parameter α=0.5π. The simulation and experimental validation show that the proposed method can effectively improve the accuracy of the localization and mapping method of the snake robot and broaden the scope of the localization and mapping method under the serpentine gait. However, this paper only investigates the SLAM adaptability of the snake robot’s motion gait in a two-dimensional plane and the application scenarios are relatively simple. The validation of the snake robot’s three-dimensional gait and more complex scenarios will be carried out at a later stage.

## Data Availability

Data are contained within the article.

## References

[1] Zhou X., Zhang Y., Qiu Z., Shan Z., Cai S., Bao G. (2024). Locomotion control of a Rigid-Soft coupled snake robot in multiple environments. Biomim. Intell. Robot..

[2] Liu J., Tong Y., Liu J. (2021). Review of Snake Robots in Constrained Environments. Robot. Auton. Syst..

[3] Borenstein J., Borrell A. The OmniTread OT-4 serpentine robot. Proceedings of the 2008 IEEE International Conference on Robotics and Automation.

[4] Liljebäck P., Pettersen K.Y., Stavdahl Ø. A snake robot with a contact force measurement system for Obstacle-Aided loco-motion. Proceedings of the 2010 IEEE International Conference on Robotics and Automation.

[5] Wright C., Buchan A., Brown B., Geist J., Schwerin M., Rollinson D., Tesch M., Choset H. Design and architecture of the unified modular snake robot. Proceedings of the 2012 IEEE International Conference on Robotics and Automation.

[6] Makhubela J.K., Zuva T., Agunbiade O.Y. A review on vision simultaneous localization and mapping (VSLAM). Proceedings of the 2018 International Conference on Intelligent and Innovative Computing Applications (ICONIC).

[7] Kohlbrecher S., von Stryk O., Meyer J., Klingauf U. A flexible and scalable SLAM system with full 3D motion estimation. Proceedings of the 2011 IEEE International Symposium on Safety, Security, and Rescue Robotics.

[8] Khan S., Wollherr D., Buss M. (2016). Modeling laser intensities for simultaneous localization and mapping. IEEE Robot. Autom. Lett..

[9] Chavan P., Murugan M., Unnikkannan E.V.V., Singh A., Phadatare P. Modular snake robot with mapping and navigation: Urban search and rescue (USAR) robot. Proceedings of the 2015 International Conference on Computing Communication Control and Automation.

[10] Ponte H., Queenan M., Gong C., Mertz C., Travers M., Enner F., Hebert M., Choset H. Visual sensing for developing autonomous behavior in snake robots. Proceedings of the 2014 IEEE International Conference on Robotics and Automation (ICRA).

[11] Nivethika S.D., Nivethetha T., Priyadharshini P., Nithyasri V.T., SenthilPandian M., Sivaprasad R. Design and development of pipe inspection snake locomotion robot. Proceedings of the 2022 International Conference on Power, Energy, Control and Transmission Systems (ICPECTS).

[12] Yang W., Bajenov A., Shen Y. (2017). Improving Low-Cost Inertial-Measurement-Unit (IMU)-Based motion tracking accuracy for a biomorphic Hyper-Redundant snake robot. Robot. Biomim..

[13] Billah M.M., Khan M.R. Smart inertial Sensor-Based navigation system for flexible snake robot. Proceedings of the 2014 IEEE International Conference on Smart Instrumentation, Measurement and Applications (ICSIMA).

[14] Gong C., Travers M., Astley H.C., Goldman D.I., Choset H. Limbless locomotors that turn in place. Proceedings of the 2015 IEEE International Conference on Robotics and Automation (ICRA).

[15] Yamada H., Mori M., Hirose S. Stabilization of the head of an undulating Snake-like robot. Proceedings of the 2007 IEEE/RSJ International Conference on Intelligent Robots and Systems.

[16] Bing Z., Cheng L., Huang K., Jiang Z., Chen G., Röhrbein F., Knoll A. Towards autonomous locomotion: Slithering gait design of a Snake-like robot for target observation and tracking. Proceedings of the 2017 IEEE/RSJ International Conference on Intelligent Robots and Systems (IROS).

[17] Wu X., Ma S. (2013). Neurally controlled steering for Collision-Free behavior of a snake robot. IEEE Trans. Control Syst. Technol..

[18] Qiao G., Song G., Zhang Y., Zhang J., Li Y. Head stabilization control for Snake-like robots during lateral undulating locomotion. Proceedings of the 2014 IEEE International Conference on Robotics and Biomimetics (ROBIO 2014).

[19] Nor N.M., Ma S. CPG-Based locomotion control of a Snake-like robot for obstacle avoidance. Proceedings of the 2014 IEEE International Conference on Robotics and Automation (ICRA).

[20] Ohno K., Nomura T., Tadokoro S. Real-Time robot trajectory estimation and 3D map construction using 3D camera. Proceedings of the 2006 IEEE/RSJ International Conference on Intelligent Robots and Systems.

[21] Liu X., Wei W., Li Y., Gao Y., Xiao Z., Lin G. (2023). Trajectory prediction and visual localization of snake robot based on BiLSTM neural network. Appl. Intell..

[22] Kim S.-J., Jin M., Suh J.-H. (2023). A study on the design of Error-Based adaptive robust RBF neural network Back-Stepping controller for 2-DOF snake robot’s head. IEEE Access.

[23] Li X., Wu H., Chen Z. Dynamic objects recognizing and masking for RGB-D SLAM. Proceedings of the 2021 4th International Conference on Intelligent Autonomous Systems (ICoIAS).

[24] Wang C., Luo B., Zhang Y., Zhao Q., Yin L., Wang W., Su X., Wang Y., Li C. (2021). DymSLAM: 4D dynamic scene reconstruction based on geometrical motion segmentation. IEEE Robot. Autom. Lett..

[25] Tesch M., Lipkin K., Brown I., Hatton R., Peck A., Rembisz J., Choset H. (2009). Parameterized and scripted gaits for modular snake robots. Adv. Robot..

[26] Servières M., Renaudin V., Dupuis A., Antigny N. (2021). Visual and Visual-Inertial SLAM: State of the Art, Classification, and Experimental Benchmarking. J. Sens..

[27] Campos C., Elvira R., Rodríguez J.J.G., Montiel J.M.M., Tardós J.D. (2021). ORB-SLAM3: An Accurate Open-Source Library for Visual, Visual–Inertial, and Multimap SLAM. IEEE Trans. Robot..

